# Hysteroscopy- and laparoscopy-based diagnosis and treatment of girls with unbroken hymen with an obstructing uterine septum: two case reports

**DOI:** 10.1186/1752-1947-8-222

**Published:** 2014-06-24

**Authors:** Songshu Xiao, Min Xue, Yajun Wan, Yueran Li, Dabao Xu

**Affiliations:** 1 Department of Gynaecology and Obstetrics, The Third Xiangya Hospital of Central South University, Changsha 410013, Hunan, China

**Keywords:** Hymen-protecting, Hysteroscopy, Laparoscopy, Obstructing uterine septum

## Abstract

**Introduction:**

Obstructing uterine septum is a rare uterine malformation. Patients with obstructing uterine septum are usually treated with laparouterotomy, causing obvious injury to both the uterus and body of the patients. Therefore, using the natural channel of the vagina is undoubtedly the best way to carry out the surgery. However, obstructing uterine septum usually occurs in puberty in girls without a history of sexual intercourse, thus iatrogenic damage to the hymen during the diagnosis and treatment cannot probably be avoided. However, Chinese people traditionally tend to use hymen intactness as a standard to judge whether an unmarried woman is chaste. Therefore, in China, to protect the hymen from damage during hysteroscopic diagnosis and treatment is of special significance for girls and women with unbroken hymens. None of the previously reported cases were treated with electrosurgical obstructing uterine septum excision based on B-ultrasound-guided hymen-protecting hysteroscopy and laparoscopy.

**Case presentation:**

Case 1 patient was a *virgo intacta* 13-year-old Chinese girl. She was admitted due to an 8-day post-menstruation lower abdominal pain. With the guidance of B-ultrasound, we observed a 30mm×20mm mixed echogenicity mass in her uterine cavity. Case 2 patient was a *virgo intacta* 14-year-old Chinese girl. She was admitted to our hospital more than 6 months after secondary dysmenorrhea and 6 days after B-ultrasound-diagnosed uterine malformations. We observed a 30mm×25mm mixed echoic area in her uterine cavity with the guidance of B-ultrasound.

Both patients were surgically treated without hymen damage with B-ultrasound-guided combined therapy of hysteroscopy and laparoscopy. A needle electrode with an 8mm diameter was placed into their uterine cavities under hysteroscopy. After obstructing uterine septum removal, their uterine cavities showed normal morphology. To protect their hymens, misoprostol was placed into their rectums to soften their cervices, so that the hysteroscope could be inserted into their cavities without damaging their hymens.

**Conclusion:**

*Virgo intacta* women with obstructing uterine septum could be treated with electrosurgical obstructing uterine septum excision based on B-ultrasound-guided hymen-protecting hysteroscopy and laparoscopy.

## Introduction

Obstructing uterine septum (OUS), also known as Robert’s uterus, is a rare obstructive malformation in the Müllerian duct of the uterus. The lower part of the diaphragm is fused with the uterine wall to close the cavity, which in turn can cause hemometra and exacerbated progressing dysmenorrhea. To date, only several studies have been carried out on OUS [1-5]. Patients with OUS were usually treated with laparouterotomy. However, transabdominal plastic surgery of the uterus will obviously cause complete fracture damage to myometrium and form a permanent scar, resulting in a risk of uterine rupture during patients’ subsequent pregnancies and childbirths. Furthermore, transabdominal laparotomy leads to a relatively large wound of the abdominal wall and causes obvious impact on the patient’s abdominal and pelvic organs. Therefore, taking advantage of the natural channel of the vagina is the best way to conduct uterine plastic surgery. However, a conventional hysteroscopy operation requires vaginal speculum distraction. Since OUS usually occurs in adolescent asexual girls, iatrogenic hymen injury will undoubtedly bring physical harm and psychological trauma to the patients, especially in China, where the hymen is of special significance for young women; hymen injury may affect the patient’s marital life in future, and even cause tragedy. Moreover, in general medical practice, physicians should also follow the basic principle of ‘no harm’. Therefore, the approach of hymen-protective hysteroscopic diagnosis and treatment in *virgo intacta* girls described in this report is of great significance.

Two *virgo intacta* patients with OUS were admitted to our hospital, and both were treated with electrosurgical OUS excision based on B-ultrasound-guided hymen-protecting hysteroscopy and laparoscopy. Here, we report these two cases.

## Case presentation

### Case 1

A 13-year-old Chinese girl was admitted to our hospital 2 days after the diagnosis of uterine mass, which was discovered due to an 8-days post-menstruation lower abdominal pain. She had menarche at the age of 11 years and had no dysmenorrhea history. Her last menstrual period started from July 16, 2010. On July 19, she showed paroxysmal left lower quadrant abdominal pain with nausea and vomiting. A computed tomography examination performed by the local hospital revealed uterine bleeding, and the results of gynecological B-ultrasonic diagnosis indicated that there was a hypoechoic mass in her uterine cavity, which looked like a 2-month-old embryo. After being admitted to our hospital, she was examined, and the results revealed a normal vulva, a large anteverted uterus, and a mass of approximately 6cm in her left uterine cavity. In addition, B-ultrasound of the urinary tract showed normal results.

To perform the surgery, at 10 p.m. 0.4mg misoprostol was placed into her rectum 3 hours before the surgery to soften her cervix [6]. On July 29, 2010, after anesthesia with intubation, she received B-ultrasound-guided hymen-protecting hysteroscopy and laparoscopy, and obstructing septal endometrium coagulation. With the guidance of B-ultrasound, we observed adenomyosis-like changes in the left side of her enlarged uterine wall, a 30mm×20mm mixed echo mass in her uterine cavity, and a 40mm thickness of her right-side uterine cavity muscle layer. The results of hysteroscopy revealed normal endocervical mucosa but a narrow angular-shaped right-side uterine cavity and a thin endometrium. One uterine horn and fallopian tube entry could be observed at the topright of her uterine cavity, but we did not observe another fallopian tube entry or other channels connected to the left side of her uterine cavity. Furthermore, the right corner of her uterine cavity showed normal morphology but the left corner was smooth and enlarged, with a 7cm diameter. In addition, her bilateral fallopian tubes and both ovaries showed normal morphology. A resectoscope with an 8mm diameter was placed into her uterine cavity under hysteroscopy, and a needle electrode was used to cut and pierce the OUS with B-ultrasound guidance to expose the endometrial tissues, with some brown fluid observed. During the process of OUS removal, we observed scattered punctate brown endometriosis lesions in the myometrium. Furthermore, using hysteroscopy, we found that the left side of her uterine cavity became smaller.

### Case 2

**Figure 1:**
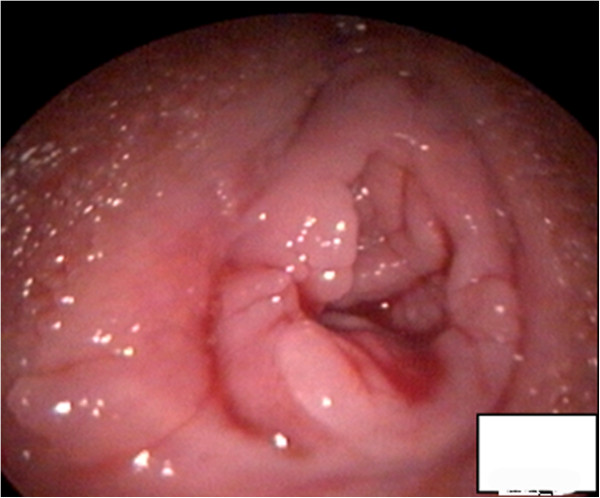
A photo showing that the hymen of the patient was not damaged after the hymen-protecting hysteroscopy.

**Figure 2:**
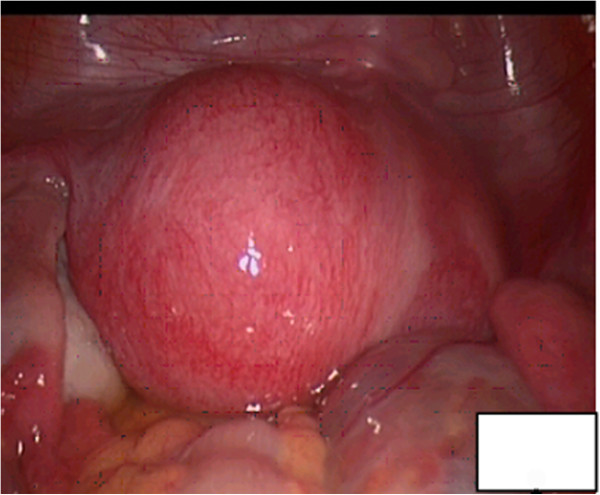
**The laparoscopy revealed a small narrow right-side uterine cavity and an enlarged left-side cavity, which was full and similar to a 40 days’ pregnancy.** The bilateral fallopian tubes and both ovaries showed normal morphology.

**Figure 3:**
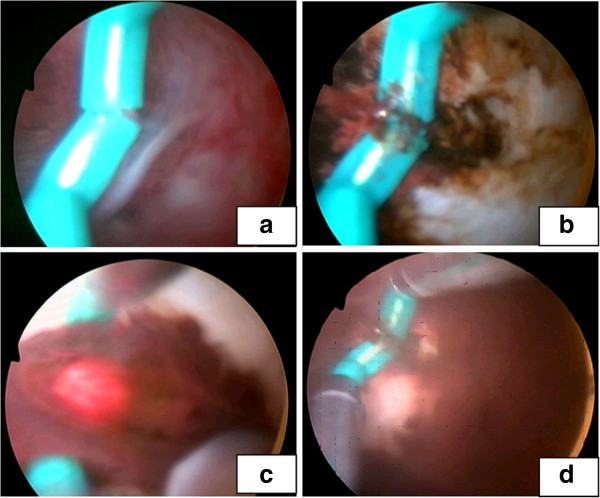
**Photos showing that a resectoscope was placed into the uterine cavity under hysteroscopy, and a needle electrode was used to cut and pierce the obstructing uterine septum with B-ultrasound guidance.** A brown blood stain on the uterine wall was observed. **(a)** Photo showing that a resectoscope was placed into the uterine cavity under hysteroscopy, and **(b)** showing that a needle electrode was used to cut and pierce the obstructing uterine septum with B-ultrasound guidance. **(c)** showing a dark brown viscous blood outflowing, **(d)** showing that a brown blood stain on the uterine wall was observed.

A 14-year-old Chinese girl was admitted to our hospital more than 6 months after secondary dysmenorrhea and 6 days after B-ultrasound-diagnosed uterine malformations. She had menarche at the age of 12 years and had a normal menstrual history. However, since January, 2010, she started to suffer from menstrual pain, which could not be alleviated until 1 week after menstruation ending. Her last menstrual period started from July 13, 2010. On July 25, the patient showed intensified persistent abdominal pain with anus bulge, which was slightly alleviated with an unclear treatment by the local hospital. She was then admitted to our hospital for further diagnosis and treatment. An anal examination revealed a slightly larger anteverted uterus with mild tenderness, and the bilateral adnexa were normal. Examination with color B-ultrasound revealed that the anteverted uterine size was approximately 47mm×37mm×30mm, and the morphology was normal; a 46mm×51mm mass was in the left side of her uterine cavity, which showed blood flow signals by color Doppler flow imaging examination and contained a 34mm×30mm echoic area. The myometrial thickness between the echoic area and the endometrium was approximately 12mm. In addition, B-ultrasound examination of her urinary tract showed normal results.B-ultrasound-guided hymen-protecting hysteroscopy and laparoscopy were performed on August 5, 2010. Misoprostol (0.4mg) was placed into her rectum at night and 3 hours before the surgery to soften her cervix. The results of hymen-protecting hysteroscopy (Figure 1) revealed a narrow horn-shaped right-side uterine cavity. One uterine horn and fallopian tube entry could be observed at the top right of her uterine cavity, but we did not observe another fallopian tube entry or other channels connected to the left side of her uterine cavity. The laparoscopy results revealed a small narrow right-side uterine cavity. However, the left side of the cavity was full without a depressed bottom, and showed an enlarged size, which was analogous to 40 days’ pregnancy (Figure 2). Her bilateral fallopian tubes and both ovaries showed normal morphology. The B-ultrasonic monitoring revealed a 30mm×25mm mixed echoic area in the left side of her enlarged uterine cavity, and the oblique septum between the bilateral cavities had a thickness of 12mm and a length of 20mm and was diagnosed as OUS. A resectoscope with an 8mm diameter was placed into her uterine cavity under hysteroscopy, a needle electrode was used to cut and pierce the OUS with B-ultrasound guidance, and subsequently a large amount of brown fluid was observed. Under hysteroscopy, her uterine wall blood was cleaned using uterine distention fluid (Figures 3a and 3d). After OUS removal, her uterine cavity showed normal morphology, with both sides of horns and fallopian tube entry visible. In addition, we found that the left side of her uterine cavity became smaller and showed normal morphology.

**Figure 4:**
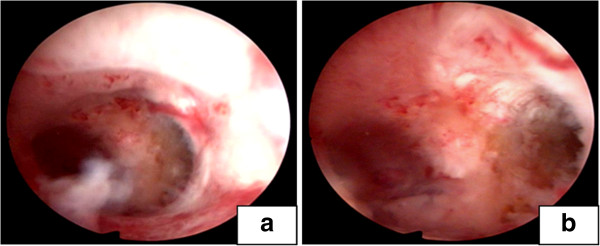
**Three months after the surgery, hysteroscopy of the case 2 patient showed the recovered uterus with normal morphology. (a)** and **(b)** photos showing that the views in the lower and upper view of uterine cavity morphology respectively.

Three months after the surgery, a hysteroscopy of the case 2 patient showed that her middle uterine wall tilted towards the intrauterine line, the bottom of her uterus protruded towards her uterine cavity similar to incomplete uterine mediastina; her left and right uterine horns were relatively deep, and both sides of uterine horns and fallopian tube entry were visible (Figures 4a and 4b). Both patients showed normal recovery after surgery. During the following 3 years of follow-up, their dysmenorrhea disappeared, and their menstruation parameters such as amount and period became normal.

## Discussion

OUS is a rare uterine malformation characterized by the moving of the intrauterine septum towards one side, which separates the uterine cavity into two, and the hemorrhage at the latching side can cause secondary dysmenorrhea [7]. OUS usually occurs after menstrual cramps in puberty in girls without a history of sexual intercourse. B-ultrasound can identify the two separated cavities, and the latching side cavity is enlarged due to the discharge failure of menstrual blood. However, B-ultrasound has its limitations. For instance, it cannot identify the uterus bicornis unicollis with one unconnected rudimentary horn.

Hysteroscopy can be used to directly observe the structure and morphology of the uterine cavity, and can firmly diagnose uterine malformations when combined with laparoscopy. In the present report, both patients were treated with B-ultrasound-guided combined therapy of hysteroscopy and laparoscopy, which can further help in the identification of a uterus bicornis unicollis with one unconnected rudimentary horn and complete uterine septum. For rare malformations, compared with the conventional open surgery and laparoscopic plastic surgery, our transvaginal surgical therapy has several advantages as follows: 1) uterine scar and trauma can be avoided; 2) the removal area and depth can be firmly determined to maximally recover the uterine cavity; and 3) the appearance and function of the uterine cavity can be protected to reduce the risk of uterine pregnancy rupture [7]. Therefore, our therapy is particularly useful for young patients with anticipated pregnancy in the future.

Furthermore, both patients were surgically treated without hymen damage. In the Chinese culture, the hymen is particularly important for young women [6]. Hence, diagnosis and treatment of uterine diseases in young women have been difficult. Routine diagnosis based on a speculum stretching the vagina always leads to iatrogenic injury to the hymen, resulting not only in physical injuries but also psychological trauma.

The difficulty in hysteroscopic examination of women with an unbroken hymen lies in the tight cervix. To protect the hymen, the cervix cannot be routinely fixed and opened, and thus it is difficult to place the hysteroscope into the uterine cavity. Hence, misoprostol was placed into the rectum of the patients to soften the cervix, so that the hysteroscope could be easily inserted into the cavity without damaging the hymen. During the surgical process, B-ultrasonic monitoring was used to enhance safety. However, the surgeon should still be experienced in hysteroscopy.

## Conclusions

OUS is a rare uterine malformation. OUS usually occurs in puberty in girls without a history of sexual intercourse. Taking advantage of the natural channel of the vagina is the best way to conduct uterine plastic surgery. However, a conventional hysteroscopy operation requires vaginal speculum distraction. Iatrogenic hymen injury will undoubtedly bring physical harm and psychological trauma to the patients, especially in China. The approach of hymen-protective hysteroscopic diagnosis and treatment in *virgo intacta* girls described in this report is of great significance. Two *virgo intacta* patients with OUS were successfully treated with electrosurgical OUS excision based on B-ultrasound-guided hymen-protecting hysteroscopy and laparoscopy.

## Consent

Written informed consent was obtained from the patients' legal guardians for publication of this case report and any accompanying images. A copy of the written consent is available for review by the Editor-in-Chief of this journal. Our local Institutional Ethics Committee approved this publication.

## Abbreviations

OUS: Obstructing uterine septum.

## Competing interests

The authors declare that they have no competing interests.

## Authors’ contributions

XM and WYJ were the main operation doctors for the patient. LYR and XDB analyzed and interpreted the patient data regarding the malformed uterus disease. XSS was a major contributor in writing the manuscript. All authors read and approved the final manuscript.
